# Time to secondary progression in patients with multiple sclerosis who were treated with first generation immunomodulating drugs

**DOI:** 10.1177/1352458512463764

**Published:** 2013-05

**Authors:** H Tedeholm, J Lycke, B Skoog, V Lisovskaja, J Hillert, C Dahle, J Fagius, S Fredrikson, A-M Landtblom, C Malmeström, C Martin, F Piehl, B Runmarker, L Stawiarz, M Vrethem, O Nerman, O Andersen

**Affiliations:** 1 Sahlgrenska University Hospital, Gothenburg, Sweden; 2 Department of Mathematical Sciences, Chalmers University of Technology and Department of Mathematical Sciences, University of Gothenburg, Sweden; 3 Karolinska University Hospital, Huddinge, Sweden; 4 Department of Clinical and Experimental Medicine/Neurology, University of Linköping, Sweden; 5 Department of Neurology, University Hospital, Uppsala, Sweden; 6 Institute of Clinical Science, Danderyds Hospital, Sweden

**Keywords:** Disease-modifying drugs, disease progression, disease severity, epidemiology, multiple sclerosis, relapsing–remitting multiple sclerosis, secondary progressive multiple sclerosis, Sweden, time to progression

## Abstract

**Background:**

It is currently unknown whether early immunomodulatory treatment in relapsing–remitting MS (RRMS) can delay the transition to secondary progression (SP).

**Objective:**

To compare the time interval from onset to SP in patients with RRMS between a contemporary cohort, treated with first generation disease modifying drugs (DMDs), and a historical control cohort.

**Methods:**

We included a cohort of contemporary RRMS patients treated with DMDs, obtained from the Swedish National MS Registry (disease onset between 1995–2004, *n* = 730) and a historical population-based incidence cohort (onset 1950–64, *n* = 186). We retrospectively analyzed the difference in time to SP, termed the “period effect” within a 12-year survival analysis, using Kaplan-Meier and Cox regression analysis.

**Results:**

We found that the “period” affected the entire severity spectrum. After adjusting for onset features, which were weaker in the contemporary material, as well as the therapy initiation time, the DMD-treated patients still exhibited a longer time to SP than the controls (hazard ratios: men, 0.32; women, 0.53).

**Conclusion:**

Our results showed there was a longer time to SP in the contemporary subjects given DMD. Our analyses suggested that this effect was not solely driven by the inclusion of benign cases, and it was at least partly due to the long-term immunomodulating therapy given.

## Introduction

First generation disease modifying drugs (DMDs) can reduce relapse frequency in relapsing–remitting multiple sclerosis (RRMS), but have shown no consistent efficacy in treating primary (PPMS) nor secondary progressive multiple sclerosis (SPMS). The debate continues about whether early and long-term administration of DMDs for treatment of the relapsing–remitting phase can induce any delay in the transition to a secondary progressive course, a transition that is expected to occur 11–19 years after initiation of the natural course of disease.^1,2^ This basic issue cannot be resolved in randomized trials, for ethical reasons.

An increasing proportion of contemporary multiple sclerosis (MS) cases have low levels of disability. Registers that were not population-based show a diminishing disability status based on the calendar year of onset,^3,4^ and an increase in age at disability.^5^ These changes are considered to have a multifactorial background, including an increased enrollment of mild cases, resulting from earlier diagnosis and increased awareness; thus, results may not prove any biological change in the course of the disease from the time of onset. A major challenge is to identify the contribution from receipt of long-term DMD therapy, without conducting randomized trials.

Several long-term follow-up (LTFU) studies were conducted after the randomized trials of first generation DMDs. These LTFUs used historical controls, reporting reduced progression to disability;^6,7^ however, the historical controls were not matched to the treated patients, plus there was considerable loss of patients at follow-up. A more stringent variant of LTFU took advantage of the conventional 2-year time lag before the onset of active therapy in the placebo group. Based on this design, studies were able to reveal a superior outcome in the original (early) treatment arms.^8,9^ “Virtual placebo” studies were able to model patients from the placebo arms, by placing them into categories, providing a design which better approached a case-control study.^10^ However, those studies had the limitation of a short follow-up and large differences among placebo groups.^11^ Another design development was to study progression to a disability milestone, immediately before and after the implementation of a community-wide DMD treatment scheme. This shortened the interval in which a large proportion of mild cases might be mixed into the study group. A therapeutic effect was shown with the Expanded Disability Status Scale (EDSS); which confirmed that the patients could be used as their own controls;^12^ however, the maximum follow-up was not much longer than that used in pivotal randomized trials. A concern with that design was the risk of regression towards the mean. Several studies demonstrated long-term effects in studies using propensity scores, a method designed to compensate for subgroup differences in non-randomized materials.^9,13,14^ Caveats proposed for propensity analyses stated that contributing factors should have predictive capacity,^15^ and that the initial pre-treatment segments of the treated material should be included in the untreated group.^16^ However, at variance with these results, a recent study based on a large database combining MS clinic data in British Columbia found that 5 years (median) of interferon beta therapy provide no significant effect on the time to reach EDSS 6 (walking with a cane).^17^

In the present study, we investigated the time interval from the onset of RRMS to the transition to SPMS in contemporary patients with MS who were being treated with DMD, as compared to historical controls. We used demographic and severity-related onset features between the groups, to adjust for differences between the groups that might be unrelated to therapy.

Our study was approved by the Research Ethics Committees of the six Swedish university hospital centers.

## Materials and methods

### Patients

**Figure 1.**
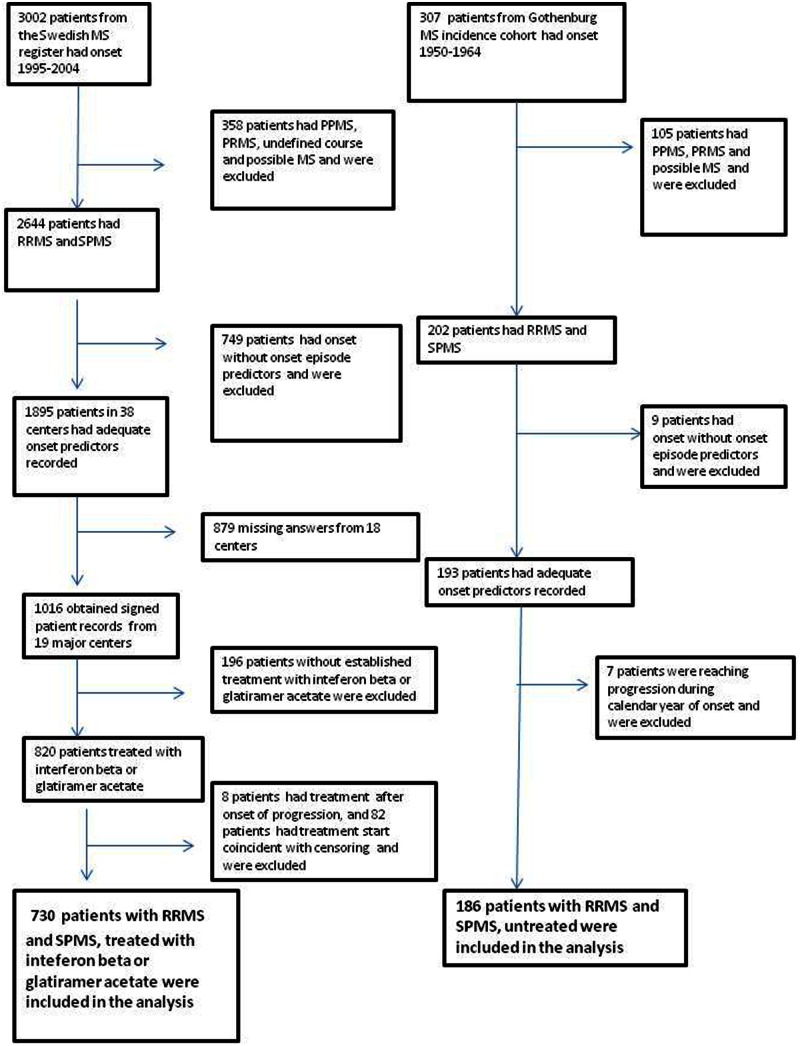
Flowchart of the steps involved in deciding patient inclusion in the study. Selections are shown for contemporary patients treated with first generation DMDs (left) and the untreated historical control individuals (right). MS: Multiple sclerosis; PPMS: primary progressive MS; RRMS: relapsing–remitting MS; SPMS: secondary progressive MS.

The untreated historical control group we used was the Gothenburg Incidence Cohort (GIC), defined as the residents of the city of Gothenburg who experienced an onset of MS during a 15-year incidence period, between 1950–1964 (*n* = 307) (Figure 1). We excluded cases with primary progression and an undefined course (*n* = 53), plus those with possible MS (*n* = 52): only patients with confirmed MS according to the Poser criteria were eligible for our study (*n* = 202). The database contained detailed individual information on relapses during a 25-year period, including three features of the relapses: “monofocal” (or not), “dominant afferent symptoms” and “complete remission” (Table 1). In the present study, we only used the values for these variables that were recorded at the onset attack. We excluded nine patients with incomplete data for these variables and seven patients who exhibited progression during the first calendar year; thus, the remaining 186 historical patients were included as the control group in the present study.

**Table 1.** Definitions of the clinical predictors: complete remission, monofocal symptoms and dominant afferent symptoms.

*Remission*	Complete remission was defined as the absence of any constant residual symptoms in the appropriate functional system, as evaluated one year after the acute phase of a relapse. Intermittent symptoms could be included.^35^ A Babinski sign was not considered sufficient to indicate incomplete remission.
*Monofocal or multifocal*	Localization of symptoms was defined on a regional basis, as determined by the proposed scheme for standardized clinical evaluation.^36^ The defined regions were: cerebral, optic nerve, brainstem and spinal cord. If symptoms and signs could only be explained by involvement of more than one region, these were defined as multifocal, but otherwise as monofocal. The requirement for multifocal lesions was stringent in the algorithm used in the GIC, as the combination of a long tract lesion and a focal lesion was never allowed to constitute a multifocal lesion.
*Afferent or efferent*	Afferent involvement was defined as symptoms or signs that could be explained by lesions in afferent tracts from the skin, muscles, eye or labyrinths (e.g. posterior columns, spinothalamic tract, vestibular nerve or optic nerve). Internuclear ophthalmoplegia and cerebellar symptoms were not included. A relapse with dominant afferent involvement was defined as afferent symptoms or signs with documented lack of major efferent symptoms such as central paresis, but a Babinski sign and increased reflexes could be included. Patients with such dominant afferent symptoms were compared with the remaining relapse patients. While a group with dominant efferent symptoms was defined in the GIC database, this was not implemented in the SMSR.

GIC: Gothenburg incidence cohort; SMSR: Swedish multiple sclerosis registry.

We retrieved data for the contemporary cohort from the Swedish MS registry (SMSR). This cohort was comprised of patients with MS, according to the Poser criteria, who had disease onset during 1995–2004; it included 1895 patients. Information on the three aforementioned MS attack features, classified with the same definitions previously used in the GIC, as well as patient gender and age at onset, was retrieved from the SMSR database. As in the historical cohort, we excluded patients with PPMS and possible MS. A further inclusion criterion was that the patient’s treating neurologists had reassessed and signed the individual SMSR records of their patients before the study. Individual registry records were sent back to 38 centers for review: of those, 19 chose to not participate. As a result of these inclusion criteria, we included SMSR data from 19 universities and other major centers for a reduced total of 1016 patients. The resulting clean file was locked to the inclusion of any more patients on 31 March 2008. An update of endpoints was performed in 2012, but 2008 was considered the censoring year.

Of the 1016 patients, we found that 820 were treated with first generation DMDs (interferon beta or glatiramer acetate). We further excluded eight patients who began treatment after the onset of secondary progression (SP), plus 82 patients who started treatment in the censoring year. Thus, a final total of 730 patients were included in the “contemporary” group receiving first generation DMDs (Figure 1). Of these patients, 76 changed their treatment to natalizumab, but this change did not occur until the last few years of their follow-up (*n* = 1 in 2005, *n* = 24 in 2006 and *n* = 51 in 2007). The patients with MS onset in the mid-1990s received delayed treatment, as compared to patients with onset after 2000, and the number of patients treated with DMDs gradually increased during the study period. A minor proportion of patients were untreated, and these were probably not representative of their category in the SMSR; therefore, we decided to restrict the comparisons of time distributions to the progression of treated patients and untreated historical controls. Patients from both cohorts were included in a 12-year survival analysis from onset to the transition to SP. The difference in time-to-endpoint between the historical and contemporary subjects was termed the “period effect.”

### Statistical methods

**Figure 2.**
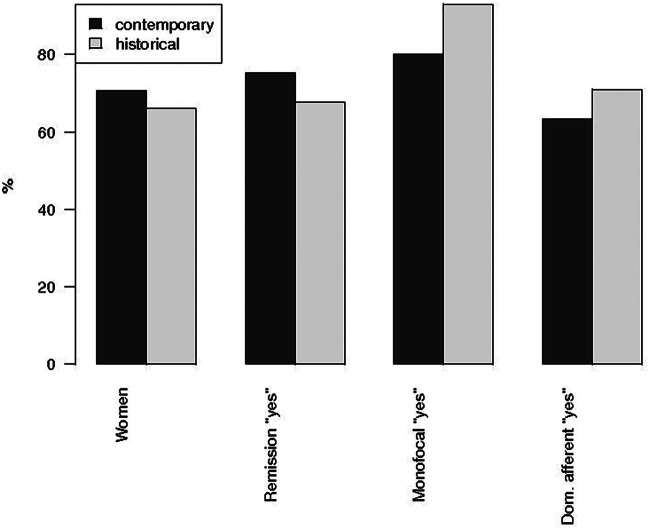
The proportions of clinical covariates and gender in the contemporary (treated, filled bars) and historical (control, open bars) cohorts.

First, we explored the data. In addition to the “period,” there were five variables of potential interest available in our collected data: gender (male/female), age at onset of disease (in years), complete remission (yes/no), monofocal onset (yes/no) and dominant afferent symptoms (yes/no). The prevalence of these covariates in both the historical and the contemporary subjects was calculated as the percentage of persons with the covariate in each group (Figure 2). Our main objective was to compare the time to SP in these two materials; thus, Kaplan-Meier estimates of this endpoint were constructed for various subgroups of the patients. To incorporate the knowledge that treated patients had not reached SP before the treatment initiation time, the treatment initiation time was left-truncated. To determine the impact that different factors had on the differences in survival, the data was stratified. To simplify the stratification, the three covariates that provided a measure of the severity of the disease (complete remission, monofocal onset and dominant afferent) were combined into a single “severity score” (0–3). These scores corresponded to the number of factors in a patient that provided an unfavorable effect on survival.^18^ For example, a patient who had an onset attack with complete remission, multifocal and efferent symptoms (Table 1), was given a severity score of 2.

After this exploratory step, several different analyses were performed with Cox proportional hazards models (i.e. Cox regression). The fit of the models was studied using a test of proportionality of hazard ratios (HRs). A measure for the explained variation was also calculated for each of the models.^19^ The final model, which was stratified by gender, included all the covariates discussed above and the number of years between the onset of the disease and the time of initiation of treatment. Calculations were mainly performed with the R package “survival” (www.r-project.org). For more details, see the Supplementary Materials.

## Results

### Patient characteristics in the two cohorts

An overview of the prevalence of the different onset factors in the two cohorts showed there was no difference in gender between the groups (70.7% females versus 66.1%, in the contemporary and historical groups, respectively; *p* = 0.245) nor in the disease onset age (average ages, 31.5 years versus 30.0 years, in the contemporary and historical cohorts, respectively; *p* = 0.581). We found that the proportion of patients with a complete remission after the first attack was higher in the contemporary cohort (75% vs. 68%, in the contemporary and historical groups, respectively; *p* = 0.04). Conversely, the proportion of patients having monofocal symptomatology at onset was higher in the historical cohort (93% versus 80% in contemporary and historical groups, respectively; *p* = 0.001). We found there was no significant difference in the proportion of patients with dominant afferent onset symptomatology (63% versus 71%, in the contemporary and historical groups, respectively; *p* = 0.149) (Figure 2). When we combined all these covariates in the “severity score,” the trends in different directions of severity did not indicate there was any marked difference in the onset composition of the contemporary and historical groups (Table 2).

**Table 2.** Frequency of the composite severity variables in the historical and contemporary groups.

Severity score	3	2	1	0
Historical	0.022	0.102	0.414	0.462
Contemporary	0.034	0.177	0.359	0.430

### Kaplan-Meier estimates of time to SP

**Figure 3.**
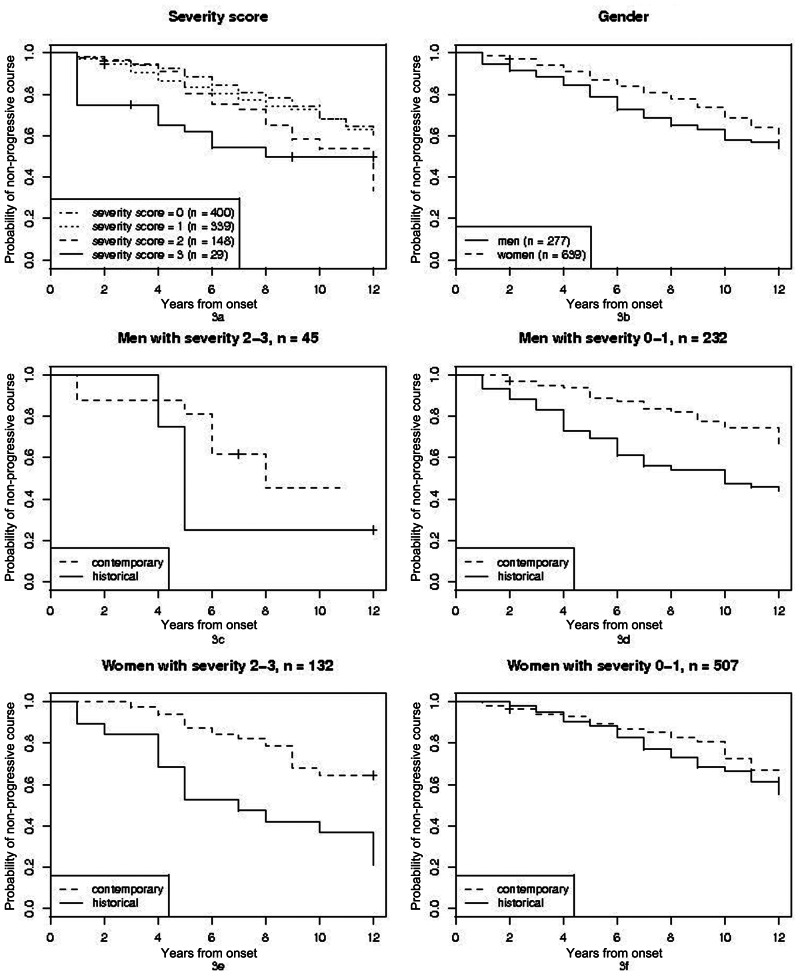
The Kaplan-Meier estimates of time to SP for stratified data, left-truncated at the treatment initiation time. Sample size in different groups is denoted by “*n*”. From upper left to lower right: stratification after severity score (3(a)), stratification after gender (3(b)), stratification after time period, gender and grouped severity score (3(c), 3(d), 3(e) and 3(f)). SP: Secondary progression.

**Figure 4.**
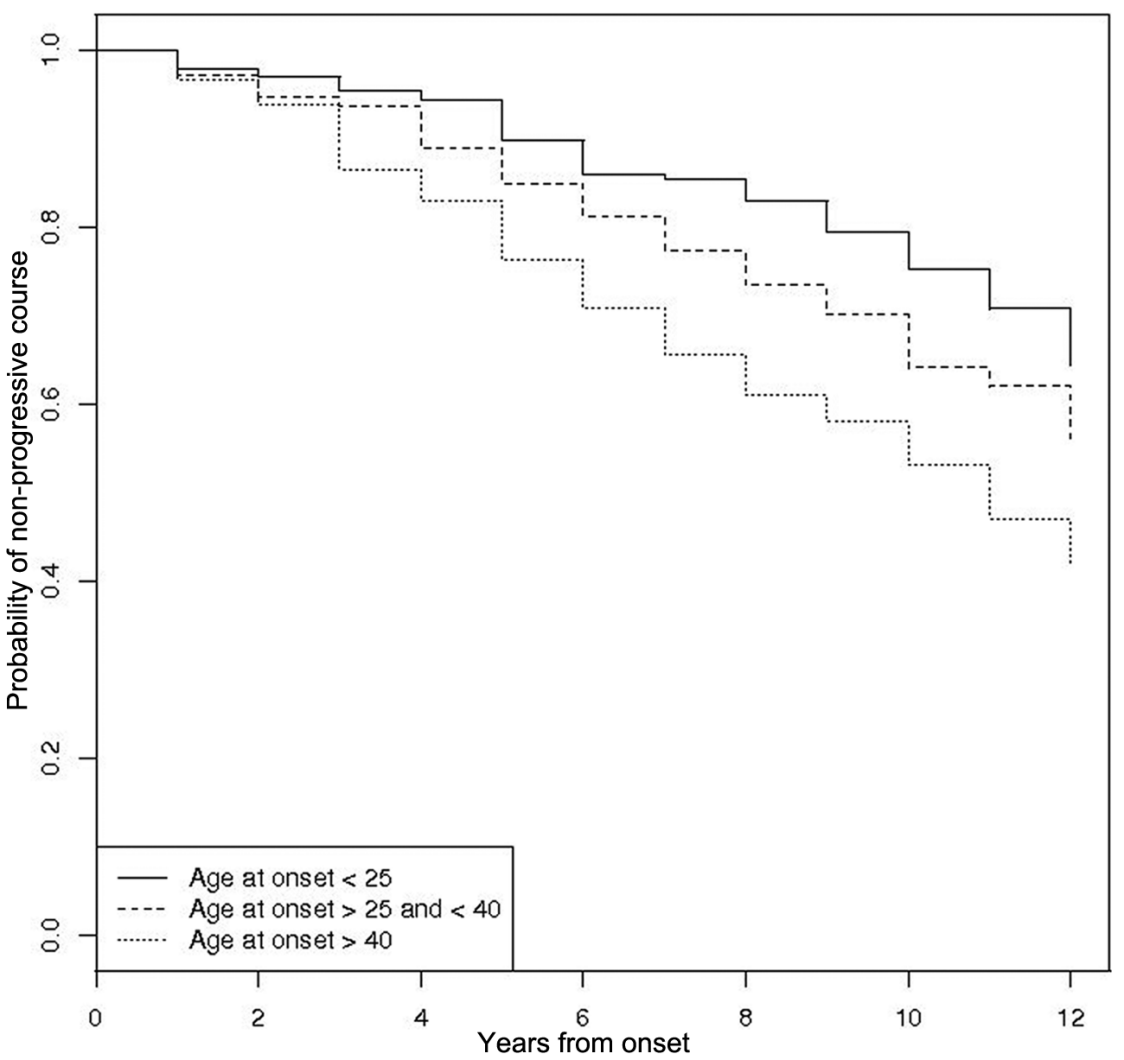
The Kaplan-Meier estimates to time to SP data, stratified after age at onset, left-truncated at the treatment initiation time. SP: Secondary progression.

Kaplan-Meier estimated survival functions for the data were performed (Figure 3 and Figure 4). Time to SP clearly decreased with an increasing age at onset (Figure 4). When our data were stratified by the severity score, as expected, the time to SP decreased with increasing severity scores, but we found the difference was marginal between scores 0 and 1 (Figure 3(a)). When the data was stratified by gender, we demonstrated that the time to SP is longer for women than for men (Fig 3(b)). The “period effect” persisted when the data was stratified by gender or severity index (we combined severity scores 0 and 1, as well as 2 and 3) (Figure 3(c-f)). Our results indicated that survival was strongly dependent on gender, age at onset and on the degree of disease severity. Moreover, the observed time “period effect” was consistently present for both genders and for all degrees of severity.

### Modeling the data with a Cox regression model

A formal analysis of the data was performed with a gender-stratified Cox regression analysis. The time to SP was modeled as a function of the onset characteristics, including: the onset age, the “period effect,” and the time from onset to the initiation of treatment (which was set to zero in the historical cohort).

First, a model was created that incorporated both genders. Although this showed a gender effect, it did not show a time-to-treatment effect; however, we found that the assumption of proportional hazards did not hold for the variables of gender and period effect. Indeed, we found indications that the effect that originated from the time “period” was dependent on the number of years after onset (for details, see Supplementary Material). Because the assumption of proportionality is vital for Cox regression, we judged this model to be unsound. Instead, we adopted two parallel models, one for men and one for women. In each of these models, the time to treatment initiation was added as a covariate. Goodness-of-fit tests revealed that the new gender-specific models did not significantly deviate from the collected data (Supplementary Materials). Royston’s measure of explained variation, similar to the conventional *R*^2^ value, was calculated for both models: We found that the Royston values were 0.24 for men and 0.11 for women.

**Figure 5.**
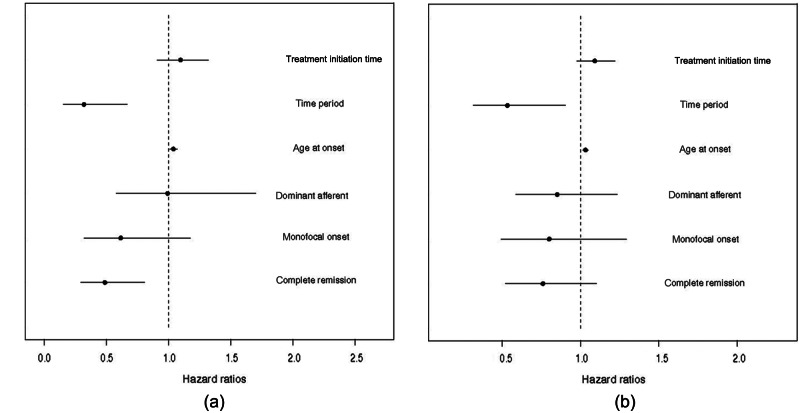
Summary of the gender-stratified Cox regression models: (a) for men, with estimated HRs of the different co-variates (Horizontal lines indicate 95% CI; (b) for women, using the same co-variates as in Figure 5(a). HR: Hazard ratio.

A summary of the two gender-specific models is presented in Table 3 and in Figure 5. The fitted coefficients in the model confirmed the results from Kaplan-Meier estimates. Both complete remission and monofocal onset had a diminishing effect on the HR, but only the effect of complete remission was found to have a significant effect (in men). The effect of age at onset was highly significant for both genders: risk increased with onset age. In addition, the HRs for the “period effect” were significant for both genders. Despite contributing to the model fit, the effect of treatment initiation time was not found to be significant; however, both estimates exhibited the “correct” sign (i.e. the longer the time to treatment, the larger the HR) and these results were remarkably alike in men and women. Despite the lack of significance, this indicated that at least part of the “period effect” was due to treatment.

**Table 3.** Summary of the gender-stratified Cox regression models, with estimated HR of the different co-variates and the corresponding *p*-values.

	Men		Women	
	HR	*p*-value	HR	*p*-value
Complete remission	0.49	0.005	0.76	0.149
Monofocal ”yes”	0.61	0.139	0.80	0.364
Dominant afferent ”yes”	0.99	0.973	0.85	0.396
Age at onset	1.04	0.008	1.03	<0.001
Treatment initiation time	1.10	0.339	1.09	0.120
Contemporary “yes”	0.32	0.002	0.53	0.020

HR: hazard ratio.

To illustrate further the estimated “period effect” on the time to SP, we calculated the 20% quantiles. The examples were calculated for four covariate profiles, all with an average age of 30 years and assuming treatment during the first year. Thus, for a man with a severity score of 0, the 20% quantile was 4 years in the historical cohort and 12 years in the contemporary cohort. For a woman with a severity score of 0, the 20% quantile was 7 years in the historical cohort and 10 years in the contemporary cohort. When the severity score was 3, we found that the 20% quantiles were, respectively, 1 and 4 years for men and 4 and 7 years for women.

## Discussion

The aim of the present study was to detect a possible difference in the time to SP between patients with RRMS during different historical periods (the “period effect”). The contemporary cohort was treated with first generation DMDs and the historical controls were untreated individuals who were identified from population-based incidence data. Our main finding was that there was a clear “period effect” on the time to SP (estimated HR 0.32 for men and 0.53 for women). We adjusted for independent demographic and clinical predictors that were derived from the database of the GIC.^20^ The aim of including those factors into the models was to make the two populations more comparable; however, the inclusion of these predictors, though they led to a more balanced comparison, may not have captured all the diversity in the populations that originated from sources other than treatment. Still, three findings supported the conjecture that the observed period effect was at least partly due to treatment:

1. The historical and contemporary patient data showed there were only minor differences at onset, as indicated by the demographic factors and features (Figure 2).
2. The “period effect” impacted the entire severity spectrum, which was represented by the strata in Figure 3. This argued against there being an effect that was confined to the most benign cases.
3. The model fit was adequate when the treatment initiation time was included in the model: There was a trend towards an increased therapeutic effect with an earlier treatment.

It is of note that the official DMD treatment indication in Sweden (≥ 2 relapses during the preceding 2-year period) favored active cases. This may contribute to the relative balance seen in the covariate compositions (Figure 2), which favored population comparability, and argued against the explanation that the period effect occurred by the inclusion of more benign cases at enrollment.^4,21^ In addition, in contrast to previous studies, we used disease onset as the baseline, instead of enrollment time; thus, our approach was less sensitive to shortened diagnostic delays.

There was no trend toward a more benign disease course in the successive three 5-year periods of the GIC: 1950–1954, 1955–1959 and 1960–1964.^20^ No change occurred in the prognosis between two prevalence materials from 1991–2000;^22^ and no change occurred in the long-term course, with onset between 1975–1995 in British Columbia, Canada.^23^ Thus, these three studies provided no evidence for a gradual, spontaneous change of MS disease towards a more benign symptomatology or trajectory during the decades before the advent of DMMs.

We used *secondary progression* (SP) as the outcome variable. The SP is responsible for nearly all severe neurological disabilities in patients with MS.^20^ The term is used according to a consensus definition.^24^ SP is required to be sustained during one year of observation. EDSS is often used as an endpoint. However, it is variable, also when expressed as 3 months sustained EDSS,^25^ and secondary progression was considered a reliable measure of an irreversible course, particularly in studies over a long span of time.^26^ Information on onset of secondary progression was re-considered if the patient reached an EDSS of 4 or 6. The EDSS 4 is often reached simultaneously with the transition to the SP.

The contemporary cohort was not strictly population-based. The national SMSR covered approximately 58% of patients during the present incidence period. Only 17 out of the 38 centers, including Gothenburg, agreed to provide us with individually reviewed and signed data. Our sample was representative of the SMSR data, in terms of the gender ratio and age. The signature requirement was aimed to ensure that the data quality was similar to that of a randomized trial; however, as it clearly favored the recruitment of patients with complete information, there was likely a “data density bias” that may have led to the loss of general representativeness. This type of bias is not uncommon in registry studies. For instance, many studies require a certain number of EDSS records for inclusion. Also, they may assign the day of a visit as the day of disability onset.^17,27,28^

We included all the available covariates in the Cox proportional hazards analysis. All covariates used were demonstrated in the GIC.^20^ The onset covariate for complete remission of the onset attack was amply confirmed in other studies.^1,2,28–34^ Other factors related to progression risk were also proposed for inclusion in the multivariate analysis, such as comorbidities, race and socioeconomic factors. Indeed, comorbidities were related to socioeconomic factors and increasing age in uncontrolled studies;^21^ however, these factors were not available in our data source. Some studies report that these factors do not have significant effects;^23^ however, admittedly, social factors may influence the inclination to seek medical care.^4^ Nevertheless, those factors were considered less relevant for our young population in Sweden, which has small differences between social levels. Data on the temporal development related to other risk factors, such as smoking and D-vitamin deficiency, were too incomplete to be included in this study.

All previous studies that investigated the long-term effect of DMDs on the onset of SP were non-randomized, with potential design flaws. These studies show a positive effect, with the exception of a recent study from British Columbia, Canada. However, the findings in the historical cohort shows a tendency in the same direction as our historical cohort, with a nearly-significant period effect (HR = 0.77; CI 0.58–1.02) in the same direction as our “period effect” (HR of 0.32 (men) and 0.53 (women)). They found an unfavorable course in their treated patients, as compared to a contemporary untreated one, with a HR = 1.30; however, the authors acknowledge that this could result from an indication bias.^17^ Had a similar indication bias been present in our treated patients, it would have resulted in an underestimated treatment effect.

There are some weaknesses in our present study. We admit that there are several critical assumptions used to motivate the statistical modelling, including the population representativity of data within the SMSR and an assumption of no in-country geographical differences. Moreover, the methods we used implicitly required that treatment decisions depend on the covariates at onset, but not on other covariates. Deviations from these assumptions may have caused biases in our study estimates and rendered the tests overly optimistic.

In conclusion, we found there was a convincing “period effect,” with a longer time to the onset of SP in the contemporarily-treated than in the historical patient data. It was not possible to completely disentangle the therapeutic effect from other factors, including the admixture of mild cases; however, we observed this “period effect” over the entire severity spectrum, and this was related to the treatment initiation time. The effect was so large that the possible inclusion of mild cases would need to be of the same magnitude as the total historical cohort. We have no incidence data to substantiate that. With a baseline of disease onset, we found no convincing evidence for a large imbalance between groups of à priori mild cases; therefore, part of the observed delay to the transition to SP was probably due to the sustained effects of long-term treatment with first generation DMDs.
